# Cricotracheal Resection in a Patient with Severe Subglottic Stenosis - Advantages of a Temporary Non-Cannulated Tracheostomy

**DOI:** 10.4274/balkanmedj.2016.0108

**Published:** 2017-03-28

**Authors:** Todor Miroslavov Popov, Tzvetomir Marinov, Julian Rangachev, Dimitar Konov, Maya Belitova

**Affiliations:** 1 Department of Otolaryngology, Medical University of Sofia, Sofia, Bulgaria; 2 Department of Anesthesiology and Intensive Care, Medical University of Sofia, Sofia, Bulgaria

**Keywords:** Cricotracheal resection, stenosis, trachea, larynx, tracheostomy

## Abstract

**Background:**

Stenosis in the area of the cricotracheal segment is still a challenging problem to be dealt with. Post-intubational cases mark an increase in recent years due to the advances in intensive care, thoracic surgery and neurosurgery departments.

**Case Report:**

This paper describes a case report of a patient with severe subglottic stenosis (grade III according to the Cotton-Myer scale), introduces a new option in cricotracheal resections - postoperative temporary non-cannulated tracheostomy and describes its advantages.

**Conclusion:**

This variation of classical operative techniques provides additional fixation of the trachea, thus relieving any transitory tension on the anastomosis; acts as a valve and decreases the air-pressure in the upper airways during coughing and sneezing in the post-operative period; and is an easy access point for video tracheoscopy of the anastomosis.

Stenosis in the area of the cricotracheal segment is still a challenging problem to be dealt with. Post-intubational cases mark an increase in recent years due to the advances in intensive care, thoracic surgery and neurosurgery departments (1). Pioneers such as Pearson, Grillo and Perelman have set the classical principles of this type of surgery (2), but new techniques and ideas are still being introduced in clinical practice (3,4).

## CASE PRESENTATION

### Patient preoperative assessment

**Figure 1:**
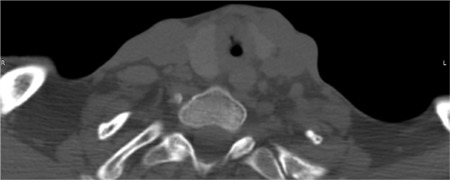
Axial computer tomography slice showing the subglottic stenosis.

A 35-year old male patient was admitted to the Department of Otorhinolaryngology with a compensated inspiratory dyspnea and biphasic stridor. Video fibroscopy revealed a severe subglottic stenosis grade III according to the Cotton-Myer scale. Computed tomography scan of the neck and chest confirmed an hourglass-shaped subglottic stenosis which carried down to the second tracheal ring (Figure 1). Three months prior to hospitalization, the patient underwent aortic valve replacement because of severe regurgitation after bacterial endocarditis. The patient was intubated in an intensive care unit for 5 days. A month after this procedure, the first signs of breathing problems began to appear. The cardiac status before our intervention displayed good functionality of the replaced valve but moderate insufficiency of the mitral and tricuspid valves, with an ejection fraction of 28%. The patient showed a decrease in functional capacity - the Duke Activity Status Index score was 4 points. According to the American Society of Anesthesiologists was grade IV. The patient's informed consent was obtained according to the guidelines of the local medical ethics committee.

### Operative procedure and postoperative period with a temporary non-cannulated tracheostomy

**Figure 2:**
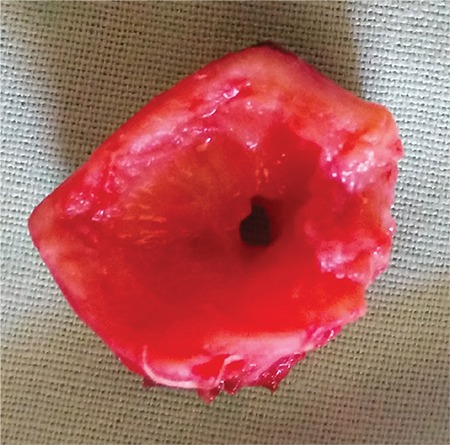
Post-operative photograph of the tracheal segment of the stenosis with a lumen of 2.5 mm.

**Figure 3:**
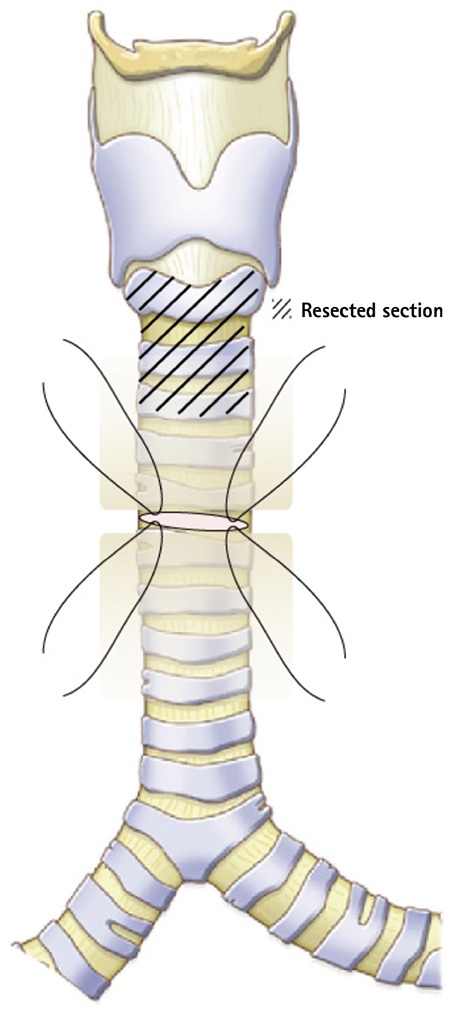
Creating a temporary non-cannulated tracheostomy. Schematic illustration.

General anesthesia was induced and patient was ventilated through a laryngeal mask airway. Low collar incision was performed and the subplatysmal flap was raised. The trachea and cricoid cartilage were dissected from the soft tissues surrounding them, while protecting the recurrent nerve. The first two tracheal rings were shrunk, with a loss of cartilaginous support. Through an incision just beneath the pathological section, the patient was intubated with a tube and afterwards the stenotic segment, the cricoid arch and the first two tracheal rings were resected. The lumen of the stenosis was 2.5 mm (Figure 2). Severe adhesions were found and resected posteriorly enveloping the corresponding segment of the esophagus. Afterwards, the trachea was released from the upper mediastinum and thyrohyoid release of the larynx was performed. Thyrotracheal anastomosis was performed using MonoPlus^®^ (B. Braun, Melsungen, Germany) (polydioxanone) sutures; the latter was verified for air leaks via laryngeal mask ventilation. At the level of the collar incision (approximately 4 cm beneath the anastomosis), an intercartilaginous incision of the trachea was performed and both superior and inferior skin flaps were sutured with the tracheal edges creating a tracheostomy, as shown in Figure 3, without placing a cannula. The postoperative period was uneventful. Crusts were removed meticulously from the tracheostomy and the latter was closed under local anesthesia at the end of the first month. One additional finding of interest was a significant post-operative increase in the ejection fraction up to 41%. The follow-up period was one year and no signs of restenosis or notable granulation were registered in the area of anastomosis or at the level of the temporary tracheostomy.

## DISCUSSION

Cricotracheal resection with primary anastomosis is an established effective treatment for subglottic and tracheal stenosis, with a reasonable success rate. Despite this, results in the published literature show a significant percentage of revision surgery and definitely not negligible unsuccessful rate in terms of decannulation. Nakache et al. (5) achieved the primary goal in 68% of cases as the decannulation rate increased to 88.5% after different types of revision surgery. Similar results are seen in the data published by Rubikas et al. (6), 86.3%, and Deckard et al. (7), 86%. Additionally, the group of Rubikas reported perioperative complications of 34.8% within 30 days of the operation in the group with cricotracheal resection.

By presenting this case, we would like to introduce a new option in cricotracheal resections - temporary non-cannulated tracheostomy. In our opinion, this addition to the classical surgical procedure gives the following advantages:

• Fixation of the trachea to the skin relieves any transitory tension on the anastomosis from the weight of the tracheobronchial tree.

• It acts as a valve which decreases the air-pressure in the upper airways during coughing and sneezing.

• It provides a very easy access point for video tracheoscopy of the anastomosis during the early post-operative period.

• Because of the lack of soft tissue between the tracheal edges and the skin flaps, placing a cannula, which could lead to secondary stenosis/granulations, is not necessary.

• There is no significant decrease in quality of life during the early post-op period since the patient breathes primarily through the upper airway and phonates almost normally.

• It acts as an easy emergency access point for ventilation if a disaster such as full anastomotic dehiscence occurs.

Negative aspects are the necessity of a second procedure to close the tracheostomy and the crusting of the stoma, which should be periodically cleaned in ambulatory settings. Prospective studies would be necessary to demonstrate the advantages of this technique.
